# Liquid Biopsy-Derived microRNAs in Pancreatic Ductal Adenocarcinoma: Matrix-Specific Evidence and Translational Challenges

**DOI:** 10.3390/ijms27125468

**Published:** 2026-06-17

**Authors:** Maria Wołyniak, Edward Zheng, Mateusz Polak, Stanisław Trojanowski, Ewa Małecka-Wojciesko

**Affiliations:** Department of Digestive Tract Diseases, Medical University of Lodz, 90-419 Lodz, Poland; maria.wolyniak@stud.umed.lodz.pl (M.W.); edward.zheng@stud.umed.lodz.pl (E.Z.); mateusz.polak@stud.umed.lodz.pl (M.P.); stanislaw.trojanowski@stud.umed.lodz.pl (S.T.)

**Keywords:** pancreatic ductal adenocarcinoma, PDAC, microRNAs, liquid biopsy, biomarker, extracellular vesicles

## Abstract

MicroRNAs are small noncoding RNA molecules that regulate gene expression at the post-transcriptional level and play a key role in cancer development, progression, and response to therapy. Their relative stability in biological fluids and disease-associated expression patterns have positioned microRNAs as promising candidates for non-invasive cancer biomarkers. Liquid biopsy enables the detection of circulating and fluid-derived microRNAs in a range of biological materials, including blood, urine, saliva, stool, pancreatic cyst fluid, and bile, offering a minimally invasive complement to tissue-based diagnostics. This approach is particularly relevant in pancreatic ductal adenocarcinoma, a malignancy with high mortality driven largely by late diagnosis, aggressive disease course, and limited opportunities for curative treatment. This review summarizes current evidence on microRNA-based liquid biopsy approaches in this cancer, with a focus on diagnostic, prognostic, and predictive relevance. Serum and plasma remain the most extensively studied sources, while urine-based microRNA profiling has shown relatively consistent diagnostic performance across available studies, including in early-stage disease. Pancreatic cyst fluid and bile offer more lesion-proximal molecular information but are limited to selected clinical scenarios because of invasive sampling requirements. In contrast, salivary microRNA signatures show greater variability and lower reproducibility across studies. Overall, liquid biopsy based on microRNA analysis shows promise as a complementary tool for pancreatic ductal adenocarcinoma detection and risk stratification. However, substantial methodological heterogeneity and limited cross-study reproducibility currently limit clinical translation, underscoring the need for standardized workflows and prospective validation of clinically relevant microRNA panels.

## 1. Introduction

MicroRNAs (miRNAs) are small, single-stranded, noncoding RNA molecules, typically 19–24 nucleotides in length, that regulate gene expression at the post-transcriptional level by binding to the 3′-untranslated region (3′ UTR) of target messenger RNAs (mRNAs) [1]. Because individual mRNAs can be regulated by multiple miRNAs and miRNA activity is context-dependent, miRNA–mRNA interactions form complex regulatory networks rather than simple one-to-one relationships [2].

MiRNAs are involved in key biological processes relevant to carcinogenesis, including tumor initiation, progression, and response to therapy. Depending on their targets, they may function either as oncogenes or tumor suppressors by modulating genes that control cell proliferation, apoptosis, differentiation, and cell-cycle control [3,4]. Their detectability in body fluids and disease-associated expression changes make them attractive candidates for biomarker development [5].

Liquid biopsy is a minimally invasive approach used to detect and analyze tumor-derived material, including circulating tumor cells (CTCs), non-coding RNAs (ncRNAs) such as miRNAs, extracellular vesicles, and proteins, in various biological fluids, most commonly blood, but also urine, saliva, and stool [6,7]. In contrast to tissue biopsy, liquid biopsy enables repeated sampling and longitudinal assessment. This approach is particularly relevant for pancreatic ductal adenocarcinoma (PDAC), where tissue access is often limited and early detection remains a major unmet clinical need.

Pancreatic cancer has one of the highest mortality-to-incidence ratios among all malignancies. Globally, it ranks lower than the most common cancers in incidence but represents a disproportionate cause of cancer-related mortality; according to GLOBOCAN 2022 estimates, it ranks 12th in incidence and 6th in cancer-related mortality worldwide [8]. The global burden of pancreatic cancer is expected to increase further over the coming decades, largely because of population ageing, population growth, and increasing prevalence of metabolic risk factors [8]. In the United States, pancreatic cancer is currently among the leading causes of cancer-related death [9]. PDAC accounts for approximately 90% of all pancreatic malignancies [10]. Most patients are diagnosed at a locally advanced or metastatic stage, and detection of a solid pancreatic tumor usually reflects clinically advanced disease and poor prognosis. The five-year survival rate remains low, and only a minority of patients are eligible for potentially curative surgical resection at the time of diagnosis [10]. Established risk factors include smoking, obesity, long-standing diabetes, chronic pancreatitis (CP), hereditary pancreatitis, family history of pancreatic cancer, and pathogenic germline variants in pancreatic cancer susceptibility genes [11,12,13]. Additional demographic and environmental contributors include older age, dietary patterns, heavy alcohol use, and occupational or environmental exposures [11]. Broad population- level screening for PDAC is not currently recommended because pancreatic cancer is relatively uncommon in the general population, and available screening tools lack sufficient accuracy, scalability, and cost-effectiveness for broad use. Current Cancer of the Pancreas Screening Consortium recommendations therefore focus on selected high-risk individuals, including those with familial pancreatic cancer kindreds or defined hereditary cancer syndromes, and generally recommend surveillance with magnetic resonance imaging/magnetic resonance cholangiopancreatography and/or endoscopic ultrasonography in experienced centers [14]. Biomarker panels that correlate with tumor presence, stage, or treatment response may nevertheless support risk stratification, longitudinal monitoring, and earlier detection in clinically defined groups.

The objective of this review is to summarize and critically compare current evidence on liquid biopsy-derived microRNAs in pancreatic ductal adenocarcinoma, with particular emphasis on their diagnostic, prognostic, and predictive relevance across different biological matrices. The working hypothesis is that the clinical performance, reproducibility, and translational applicability of microRNA biomarkers depend strongly on the biological material analyzed. Specifically, blood- and urine-based microRNA assays may offer the greatest feasibility for non-invasive detection and longitudinal monitoring, whereas pancreatic cyst fluid, bile, and pancreatic juice may provide higher biological specificity but more limited clinical applicability because of invasive sampling requirements.

## 2. Literature Search Strategy and Study Selection

A structured literature search was performed primarily using the PubMed database to identify studies evaluating microRNAs in liquid biopsy-derived biological materials in pancreatic ductal adenocarcinoma. The search was last updated on 14 April 2026, and articles published up to that date were considered eligible. The following search terms were used alone and in combination: “pancreatic cancer”, “pancreatic ductal adenocarcinoma”, “microRNA”, “miRNA”, “liquid biopsy”, “serum”, “plasma”, “urine”, “saliva”, “stool”, “feces”, “bile”, “pancreatic juice”, “pancreatic cyst fluid”, “exosome”, “extracellular vesicles”, “diagnosis”, “prognosis”, “predictive biomarker”, “chemoresistance”, and “treatment response”. The main PubMed search query was: (“pancreatic cancer” OR “pancreatic ductal adenocarcinoma” OR PDAC) AND (microRNA OR miRNA OR miRNAs) AND (“liquid biopsy” OR serum OR plasma OR urine OR saliva OR stool OR feces OR bile OR “pancreatic juice” OR “pancreatic cyst fluid” OR exosome OR “extracellular vesicles”). Additional relevant articles were identified by screening the reference lists of eligible studies.

Studies were included if they were original research articles evaluating microRNA expression in human liquid biopsy-derived samples from patients with pancreatic ductal adenocarcinoma or pancreatic cystic lesions with malignant potential, and if they reported diagnostic, prognostic, predictive, or biologically relevant outcomes. Reviews, editorials, conference abstracts without full text, non-human studies, tissue-only diagnostic studies, and articles lacking sufficient methodological detail or quantitative microRNA data were excluded. Selected preclinical studies were considered only in the treatment-response section when they provided mechanistic evidence on microRNA-mediated chemotherapy or radiotherapy resistance. Overall, 90 studies were included in the qualitative synthesis. This manuscript was designed as a structured narrative review; therefore, no formal risk-of-bias assessment or meta-analysis was performed.

## 3. Liquid Biopsy-Derived MicroRNAs by Biological Matrix

**Figure 1:**
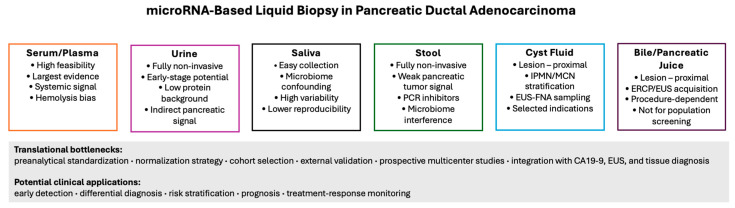
Matrix-specific framework for microRNA-based liquid biopsy in pancreatic ductal adenocarcinoma. The figure summarizes the main biological matrices discussed in this review and highlights their relative advantages, limitations, and potential clinical applications. Blood and urine offer the greatest feasibility for non-invasive diagnostic validation, whereas pancreatic cyst fluid, bile, and pancreatic juice provide more lesion-proximal molecular information but require invasive or procedure-dependent sampling. Across all matrices, clinical translation is limited by preanalytical variability, heterogeneous normalization strategies, differences in control cohorts, and insufficient prospective validation.

In recent years, miRNAs have been extensively investigated as biomarker candidates in pancreatic cancer. Across the included studies, these molecules were evaluated for multiple applications, including early detection, disease monitoring, and prognostic stratification. Despite this growing body of evidence, no miRNA panel has yet achieved universal clinical implementation. The reviewed studies highlight several factors that critically influence panel performance, particularly the choice of biological matrix and the analytical form of miRNAs, whether assessed as free-circulating molecules or as extracellular vesicle-associated cargo. The main biological matrices investigated for miRNA-based PDAC detection are summarized in Figure 1. Among the included publications, the majority (78 of 90) evaluated free-circulating miRNAs isolated directly from biological fluids, whereas only a minority focused on extracellular vesicle–associated or exosome-derived miRNAs. Extracellular vesicles comprise heterogeneous vesicle populations, and exosomes represent only one subtype within this broader category. In the reviewed studies, exosome-associated microRNAs generally referred to microRNAs isolated from small extracellular vesicle–enriched fractions; however, terminology, isolation protocols, and vesicle characterization differed across studies. Therefore, exosome-associated findings should be interpreted cautiously.

### 3.1. Blood-Derived microRNAs

Serum and plasma were the most frequently investigated matrices, reported in 43 and 32 studies, respectively, reflecting their accessibility and compatibility with established analytical workflows. Several studies noted matrix-dependent differences in microRNA detectability. Higher total RNA yield and a broader detectable microRNA spectrum were reported in serum compared with matched plasma samples using RT-qPCR [15]. This observation was attributed to coagulation-related cellular activation or lysis during serum preparation, leading to the release of intracellular RNAs [15]. Such effects may influence microRNA detectability; however, their impact on longitudinal measurements has not been systematically evaluated. Across the reviewed studies, multiple plasma- and serum-derived microRNA panels demonstrated diagnostic accuracy exceeding that of CA19-9, the most widely used clinical biomarker for pancreatic diseases.

A three-miRNA panel was analyzed in 142 plasma samples from individuals with and without PDAC using RT-PCR to validate its diagnostic value [16]. All three miRNAs, miR-125a-3p, miR-4530, and miR-92a-2-5p, were significantly upregulated in affected patients. The reported AUC values for the individual miRNAs ranged from 0.850 to 0.910, indicating strong diagnostic discrimination compared to single-marker analyses [16]. Plasma-derived exosomal miRNAs were evaluated in a cohort of 18 individuals with RT-qPCR [17]. A four-miRNA panel comprising miR-93-5p, miR-339-3p, miR-425-5p, and miR-425-3p was identified as differentially expressed between affected patients and healthy controls. The panel achieved an AUC of 0.885, with a sensitivity of 80% and specificity of 94.7%, demonstrating discriminatory ability comparable to CA19-9 [17]. Elevated expression of miR-22-3p, miR-642b-3p, and miR-885-5p in combination with CA19-9 was reported in a cohort of 35 patients with pancreatic cancer, including individuals diagnosed at early clinical stages (IB and IIB) [18]. Serum samples from 303 patients with PDAC and 760 controls were analyzed to assess the diagnostic accuracy of miR-25 [19]. Quantification via qRT-PCR demonstrated that miR-25 effectively discriminated cancer patients from healthy individuals, with an AUC of 0.915 [19]. Integrated analyses of tumor tissue and serum-derived exosomes identified six miRNAs (let-7b-5p, miR-192-5p, miR-19a-3p, miR-19b-3p, miR-223-3p, and miR-25-3p) as significantly upregulated in patients with the disease. A composite panel based on these miRNAs achieved an AUC of 0.910. Elevated serum levels of miR-19a-3p were additionally associated with reduced overall survival, with patients in the high-expression group showing a markedly shorter median OS compared with the low-expression group [20]. Taken together, these studies suggest that blood-derived miRNA panels may improve diagnostic discrimination compared with single circulating markers. Nevertheless, most blood-based studies remain limited by case–control design, heterogeneous control cohorts, relatively small validation sets, and inconsistent normalization strategies. Direct comparison between serum, plasma, and extracellular vesicle–enriched fractions is further limited by differences in preanalytical handling, RNA isolation protocols, and analytical platforms. These limitations indicate that blood-derived microRNA panels should be interpreted as promising candidates rather than clinically validated diagnostic tools.

### 3.2. Urinary and Salivary microRNAs

Urine and saliva have been explored as non-invasive sources of miRNA biomarkers. Urine may offer analytical advantages due to lower protein content, potentially reducing interference during extraction, whereas saliva provides an easily accessible matrix with molecular exchange with the systemic circulation.

The first effort to identify urinary miRNA biomarkers for this malignancy demonstrated that miR-30e, miR-143, and miR-223 were markedly overexpressed in the urine of patients with PDAC across all disease stages and in patients with chronic pancreatitis compared with healthy controls [21]. CP is a critical comparator because inflammatory pancreatic masses may mimic malignancy and may therefore lead to unnecessary surgery. The presence of focal CP among resected solid pancreatic lesions underscores the need for reliable preoperative differentiation [22]. In the same urinary biomarker study, miR-223 and miR-204 significantly differentiated stage I disease from CP, with higher expression in early-stage cancer. miR-143, miR-223, and miR-30e were also upregulated in stage I disease compared with healthy controls, whereas miR-143, miR-23, and miR-204 were higher in stage I than in stages II–IV. AUC was assessed only for stage I PDAC versus healthy controls, with the best single-marker performance for miR-143 (0.862) and the best overall result for miR-143 + miR-30e (0.923; *p* = 0.04 vs. miR-143 alone) [21]. Consistent with these findings, miR-1246 was identified as a promising biomarker, demonstrating significantly elevated expression in urine and serum, but not in saliva, from patients with PDAC compared with healthy controls [23]. In particular, urinary miR-1246 showed high diagnostic accuracy, with an AUC of 0.90, sensitivity 90.2% and specificity 83.3% [23]. The urinary exosomal miR-3940-5p/miR-8069 ratio was also significantly higher in PDAC than in both healthy controls and CP (median 1.07 vs. 0.47 and 0.54, respectively; both *p* < 0.001), indicating potential value in differential diagnosis [24]. ROC analysis yielded an AUC of 0.732, and although the ratio alone had moderate sensitivity (58.1%), its combination with CA19-9 increased sensitivity to 93.0% [24]. The signal also tended to be stronger in urine than in serum exosomes, suggesting that urine may be a useful non-invasive source of diagnostic exosomal miRNAs [24]. More recently, additional urinary miRNAs, including miR-744-5p, miR-572, miR-210-3p, and miR-575, were evaluated and found to be upregulated in patients with PDAC; however, only miR-210-3p reached statistical significance compared with healthy controls (*p* = 0.009) [25]. Importantly, these miRNAs should not be interpreted as disease-specific markers. Similarly, the remaining urinary candidates require interpretation as part of disease-specific panels rather than as standalone biomarkers, because individual miRNAs may participate in common oncogenic or stress-response pathways across different diseases [3,4,25].

Collectively, these findings support the potential role of urinary miRNAs, especially those detectable in early-stage disease, as components of future diagnostic panels. The relatively stronger internal consistency of urine-based miRNA studies may partly reflect several factors: protection of miRNAs within extracellular vesicles, lower analytical background in urine compared with blood, and reduced susceptibility to local confounders than saliva, such as oral inflammation, microbiome composition, and collection-related variability. Although these mechanisms remain incompletely understood, they may help explain why urinary miRNA profiling has so far shown more reproducible results and more promising early-stage PDAC performance than salivary miRNA-based approaches.

In contrast, salivary miRNAs have been less extensively studied and show greater variability across reports. miR-21 and miR-34a were identified as significantly upregulated in both saliva and serum from patients with PDAC, with good diagnostic performance reflected by AUC values of 0.889 and 0.865, respectively [26]. A more comprehensive analysis evaluated salivary miRNA expression not only in patients with PDAC, but also in individuals with acute or chronic pancreatitis and other non-pancreatic gastrointestinal diseases [27]. In that study, salivary hsa-miR-21, hsa-miR-23a, hsa-miR-23b, and miR-29c were consistently upregulated across multiple PDAC stages and demonstrated high specificity compared with healthy controls [27].

These salivary miRNAs should not be interpreted as pancreas-specific markers. miR-21, miR-23a, miR-23b, and miR-29c have been implicated in several malignancies and inflammatory conditions, where they may regulate apoptosis, proliferation, epithelial–mesenchymal transition, fibrosis, immune responses, and extracellular matrix remodeling. Their detection in saliva therefore most likely reflects a mixed biological signal rather than a tumor-specific pancreatic source, supporting the need for disease-specific panels and carefully selected control groups [3,4,26,27].

Preanalytical standardization is particularly important for salivary miRNA analysis. Saliva collection should be performed under controlled conditions, preferably before eating, drinking, smoking, or oral hygiene procedures, and samples should be processed or stabilized promptly to limit RNA degradation and variability related to oral contamination, cellular debris, and microbiome composition. However, collection volume, processing time, centrifugation protocol, and storage conditions differ between studies, which contributes to limited reproducibility and complicates direct comparison of reported salivary miRNA signatures [26,27,28,29].

Importantly, hsa-miR-23a and hsa-miR-23b were also significantly overexpressed in pancreatic precursor lesions, including intraductal papillary mucinous neoplasms (IPMNs) and pancreatic intraepithelial neoplasia, suggesting a potential role of salivary miRNAs in detecting early neoplastic transformation rather than advanced disease alone [27]. However, despite these encouraging observations, salivary miRNA signatures remain less reproducible across studies, and reported performance metrics vary substantially depending on cohort composition and selected control populations [26,27,28,29].

Additional evidence from a Japanese cohort demonstrated overexpression of miR-1246, miR-3976, miR-4306, and miR-4644 in salivary samples from patients with PDAC compared to healthy controls. Among these, miR-1246 and miR-4644 showed notable diagnostic value and discriminatory power, with AUC values of 0.814 (*p* = 0.008) and 0.763 (*p* = 0.026), respectively [28]. Significant downregulation of miR-3679-5p and upregulation of miR-940 were observed in saliva from patients with PDAC compared with healthy controls. Both miRNAs demonstrated high sensitivity, 82.5% for miR-3679-5p and 90% for miR-940, though their specificity was lower, at 45% and 40%, respectively [29].

The lower specificity of salivary miRNA signatures may reflect the fact that saliva is influenced not only by systemic tumor-derived signals but also by local oral conditions, including inflammation, periodontal disease, oral microbiota, food intake, salivary gland secretion, and collection-related variability. Therefore, salivary miRNA profiles should not be interpreted as direct equivalents of tumor tissue or blood-derived profiles. Some miRNAs may overlap between saliva, blood, and tumor tissue, but their relative abundance, carrier form, and biological origin may differ substantially between compartments. In saliva, detected miRNAs likely represent a mixed biological signal derived from local oral sources, systemic circulation, extracellular vesicles, and salivary gland secretion. These preanalytical and biological factors may partly explain the weaker reproducibility and lower specificity of salivary miRNA studies compared with urine- or blood-based approaches [26,27,28,29].

Despite relatively high sensitivity, the low specificity reported in this study limits the clinical utility of these salivary miRNAs. Taken together, current evidence suggests that urine represents a more robust and reproducible non-invasive matrix for miRNA-based PDAC detection, particularly in early-stage disease, whereas salivary miRNAs remain exploratory and require further validation in larger, well-controlled cohorts.

### 3.3. Pancreatic Cyst Fluid microRNAs

With the increasing use of cross-sectional imaging, pancreatic cystic lesions are detected with growing frequency, raising concern regarding their clinical significance and malignant potential [30]. However, the absolute risk of malignant transformation remains low. In the United States, only 1137 malignant cases have been reported among approximately 3.43 million pancreatic cystic lesions, corresponding to an estimated incidence of 0.03% [30]. As a result, routine surgical resection of all detected cysts is not feasible, given the high prevalence of benign lesions and the substantial morbidity and mortality associated with pancreatic surgery [31].

Pancreatic cystic lesions are broadly classified according to pathological features and malignancy risk. These include inflammatory pseudocysts, serous cystadenomas, and mucinous cystic neoplasms (MCNs), as well as less common entities such as cystic pancreatic neuroendocrine tumors and solid pseudopapillary neoplasms [31]. Mucinous cystic neoplasms and IPMNs are clinically important because they can progress to invasive cancer [32,33]. IPMNs and conventional PDAC differ in their clinicopathological development. IPMNs arise as intraductal mucin-producing epithelial neoplasms and may progress through graded dysplasia to invasive carcinoma, whereas conventional PDAC most often develops from microscopic precursor lesions, particularly pancreatic intraepithelial neoplasia [32,33]. Although both processes may involve shared oncogenic pathways, including *KRAS*-related alterations, IPMN progression is linked to cystic intraductal epithelial transformation, while conventional PDAC is typically characterized by early stromal invasion and late clinical detection [10,32,33]. IPMNs are further categorized according to ductal involvement, and this distinction has direct clinical relevance [32]. Main-duct IPMNs demonstrate substantially higher malignant potential, with invasive carcinoma present in approximately 62% of cases at surgical resection, compared with 24% in branch-duct lesions [32]. Less common cystic entities may also harbor malignant potential, although their prevalence and risk profiles differ substantially [31].

For clinical interpretation, pancreatic cystic lesions may be grouped according to malignant potential into low-risk, high-risk, and malignant categories [31,32]. Low-risk lesions include pseudocysts and serous cystadenomas, whereas high-risk lesions comprise MCNs and IPMNs [31,34]. Conventional imaging modalities such as computed tomography and magnetic resonance imaging are essential for detection and morphological assessment, but their accuracy for risk stratification remains limited [31,32]. In pancreatic cystic lesions, imaging findings are integrated with clinical features, endoscopic ultrasonography, cytology, and cyst fluid biomarkers, but definitive diagnosis of malignancy ultimately relies on cytological or histopathological confirmation when available [31,32]. Consequently, endoscopic ultrasonography (EUS) with fine-needle aspiration (EUS-FNA) is frequently employed to enable cyst fluid analysis in selected cases [31,35]. Currently used cyst fluid biomarkers include carcinoembryonic antigen (CEA), amylase, and in some cases glucose. A CEA concentration >192 ng/mL is commonly used to suggest mucinous etiology, whereas low cyst fluid glucose has been explored as an adjunctive marker for differentiating mucinous from non-mucinous pancreatic cystic lesions [31,35]. These markers are used for cyst characterization rather than for monitoring pancreatic cancer before, during, or after chemotherapy. Despite their clinical utility, these markers lack sufficient accuracy to reliably predict malignant transformation, contributing to ongoing diagnostic uncertainty and potential overtreatment [31,32,35]. In this context, EUS-guided tissue acquisition remains an important part of diagnostic confirmation and biomarker development. EUS-FNA and EUS-FNB provide cytological or histological material, whereas liquid biopsy-derived microRNAs may provide complementary molecular information through repeated or lesion-proximal sampling. Therefore, microRNA-based liquid biopsy should be evaluated as an adjunct to imaging, EUS-guided tissue acquisition, cytology, histopathology, CA19-9, and molecular testing rather than as a replacement for established diagnostic pathways [31,35].

Emerging evidence indicates that miRNA profiling of pancreatic cyst fluid obtained via EUS-FNA or ERCP may provide diagnostically relevant information beyond conventional cyst fluid markers [36]. This approach leverages the fact that cyst fluid contains miRNAs released directly from the epithelial lining of the lesion. The cells undergoing neoplastic transformation are primarily dysplastic epithelial cells lining mucinous cystic lesions, particularly IPMNs and MCNs, which may progress toward invasive carcinoma [32,33]. In contrast, circulating miRNAs detected in serum reflect a broader and more heterogeneous systemic pool [6,15]. Because cyst fluid is in direct contact with the epithelial lining of the lesion, cyst-derived miRNA profiles may provide a more lesion-proximal signal than circulating serum or plasma profiles [35,36,37,38,39,40]. This provides a biological rationale for potentially higher lesion specificity, but does not by itself establish clinical specificity. The latter is supported only indirectly by studies showing differential expression of selected cyst-fluid miRNAs in mucinous, premalignant, or malignant pancreatic cystic lesions, including miR-200b, miR-21, miR-221, miR-216, and miR-17-3p [35,36,37,38,39,40]. Therefore, cyst-fluid miRNAs should currently be interpreted as complementary molecular markers for cyst characterization rather than validated stand-alone diagnostic tests.

Several independent studies have identified specific cyst-fluid miRNAs associated with high-risk or mucinous pancreatic lesions. Increased expression of miR-21, miR-221, miR-200b, miR-216, and miR-17-3p has been repeatedly reported in cysts with premalignant or malignant features [35,36,37,38,39,40]. Among these, miR-200b has been particularly well characterized. Absolute quantification of miR-200b in pancreatic cyst fluid enabled reliable discrimination between mucinous and non-mucinous cystic neoplasms, achieving a sensitivity of 83.3%, specificity of 90.9%, and an AUC of 0.886 (*p* = 0.002) [35]. Interestingly, miR-24 demonstrated similar discriminatory performance, suggesting that both markers may be of diagnostic value in this setting [35]. Complementary findings were reported for miR-21, which showed higher expression in mucinous cysts—including IPMNs and mucinous cystic neoplasms—compared with non-mucinous lesions (mean fold change 7.0; *p* < 0.01). Receiver operating characteristic analysis indicated a sensitivity of 80% and specificity of 76%, supporting the potential utility of miR-21 as an adjunctive marker for cyst risk stratification [40]. Taken together, these findings suggest that cyst-fluid miRNA profiling may represent a complementary molecular approach for the characterization of pancreatic cystic lesions, particularly in selected cases in which imaging, cytology, cyst fluid carcinoembryonic antigen, glucose, amylase, or mutation-based analyses do not provide sufficient diagnostic certainty. However, available evidence remains based on limited cohorts and selected candidate miRNAs; therefore, further prospective validation and analytical standardization are required before cyst-fluid miRNA profiling can be incorporated into routine clinical decision-making.

### 3.4. Stool-, Bile-, and Pancreatic Juice-Derived microRNAs

Early investigations of stool-derived miRNAs have primarily focused on gastrointestinal conditions such as inflammatory bowel disease (IBD) and colorectal cancer (CRC) [41,42,43,44]. Owing to variable gastrointestinal transit times and exposure to enzymatic degradation, particularly RNase activity, extracellular vesicle–associated miRNAs, including exosomal miRNAs, may retain greater stability and therefore appear more suitable for potential clinical application in fecal material [45,46].

Stool represents a completely non-invasive sample type, and miRNAs can remain stable if extraction protocols are appropriately optimized [44,45,46]. In addition, fecal miRNA profiles may reflect interactions between pancreatic tumors, pancreatic exocrine secretion, and the gut microbiome [47,48,49]. However, pancreatic-derived miRNAs are present in stool at very low concentrations, resulting in substantial signal dilution [47,48,49]. Stool composition varies widely depending on diet, consistency, and transit time, and contains multiple PCR inhibitors and RNA-degrading substances, all of which complicate analytical reliability [44,45,46,47,48]. Nevertheless, pancreatic juice is secreted into the intestinal lumen, providing a biological rationale for detecting pancreatic cancer–associated miRNAs in fecal material [47,48,49].

Methodologically, fecal miRNA analysis requires careful interpretation. MiRNAs detected in stool may include extracellular vesicle–associated and non-vesicular fractions; therefore, exosome-associated miRNAs should not be interpreted as the only detectable fraction [44,45,46]. Fecal sample collection and processing are less reproducible than blood- or urine-based workflows because stool composition is influenced by diet, intestinal transit time, hydration, microbiome composition, pancreatic exocrine function, sample consistency, storage conditions, and the presence of PCR inhibitors. For this reason, identification of fecal miRNA profiles should be performed using standardized collection, stabilization, extraction, and normalization protocols, ideally with prompt freezing or RNA stabilization and predefined quality-control criteria [44,45,46,47,48]. In principle, bacterial small non-coding RNAs and human miRNAs may be distinguished bioinformatically by sequence alignment and species-specific annotation, although this issue has not been systematically addressed in most PDAC fecal miRNA studies. Nevertheless, assigning a fecal miRNA signal specifically to a pancreatic tumor remains challenging; in most studies, pancreatic origin is inferred indirectly from differential expression between PDAC, chronic pancreatitis, and control groups, rather than proven by direct molecular tracing [47,48,49].

Only a limited number of studies have evaluated fecal miRNAs in PDAC. Significantly higher relative abundance of miR-181b, miR-196a, and miR-210 was reported in stool samples from patients with PDAC compared with healthy controls (*p* = 0.033, *p* = 0.043, and *p* = 0.011, respectively) [47]. In receiver operating characteristic analysis, miR-181b and miR-210 discriminated PDAC from healthy controls with AUC values of 0.745 and 0.772, respectively [47]. For miR-181b, sensitivity and specificity were 84.6% and 51.7%, respectively, whereas for miR-210, they were 84.6% and 65.5%, respectively [47]. Notably, miR-196a expression correlated with maximum tumor diameter (Spearman r = 0.516, *p* = 0.041), suggesting a potential association with tumor burden [47]. These findings indicate that fecal miR-181b and miR-210 may have value as accessible adjunct biomarkers for distinguishing PDAC from healthy controls, whereas miR-196a may be more closely related to tumor burden than to diagnostic discrimination [47].

Even low-abundance miRNAs, including miR-216a, can be consistently quantified in fecal samples [48]. In one study, the expression levels of miR-196a, miR-216a, miR-143, and miR-155 were highest in healthy controls, lower in CP, and lowest in patients with PDAC, indicating a stepwise decrease across the analyzed groups. A combined panel showed better discriminatory performance than individual miRNAs alone. At the same time, despite their relative stability in stool, fecal miRNA profiles may not fully reflect tumor tissue biology, possibly because pancreatic fluid outflow and exocrine pancreatic function are impaired in PDAC [48]. In contrast, significantly increased fecal expression of miR-155 and miR-21 was reported in patients with PDAC compared with individuals with CP [49]. In this study, a two-miRNA panel consisting of miR-21 and miR-155 achieved high sensitivity (93.33%), while inclusion of miR-216 yielded a three-miRNA panel with balanced diagnostic performance (AUC 0.8667; sensitivity and specificity both 83.33%) [49]. Notably, the available studies do not show a consistent direction of change for all candidate fecal miRNAs. This is particularly evident for miR-155, which was reported as decreased in PDAC in one study, but increased in another, underscoring the limited reproducibility of fecal miRNA findings across cohorts [48,49].

Several studies have also examined miRNAs in bile and pancreatic juice collected during endoscopic procedures such as EUS or endoscopic retrograde cholangiopancreatography (ERCP). Bile-derived miRNAs have been analyzed in samples obtained during ERCP, whereas extracellular vesicle-associated miRNAs from pancreatic juice have been evaluated in pancreatic juice collected after secretin stimulation during EUS [50,51]. These materials are therefore lesion-proximal but procedure-dependent, which limits their applicability to selected clinical scenarios rather than population-level screening. Bile-derived miRNAs, including miR-10b, miR-155, miR-106b, miR-30c, and miR-212, were reported to discriminate PDAC from benign conditions [50]. Extracellular vesicle–associated miRNAs from pancreatic juice obtained after secretin stimulation during EUS have also been evaluated. A composite panel incorporating pancreatic juice miR-21, miR-25, miR-16, serum miR-210, and CA19-9 differentiated PDAC cases from controls with a sensitivity of 84.2% and specificity of 81.5% [51]. More recently, the MIRABILE study identified bile miR-182-5p as significantly upregulated and miR-340-5p as significantly downregulated in malignant compared with benign samples [52].

Overall, stool-, bile-, and pancreatic juice-derived miRNAs offer attractive biological and practical features, particularly non-invasive sampling for stool and lesion proximity for bile and pancreatic juice. However, substantial analytical variability, low signal abundance, and reliance on invasive acquisition for bile and pancreatic juice currently limit their application to adjunctive roles in selected clinical contexts rather than routine screening. The key advantages, limitations, and representative references for each biological matrix are summarized in Table 1.

### 3.5. MicroRNAs and Treatment Response

Current pharmacological management of PDAC relies primarily on conventional chemotherapy regimens, including FOLFIRINOX, a combination of oxaliplatin, leucovorin, irinotecan, and 5-fluorouracil, and gemcitabine, administered alone or in combination with nab-paclitaxel, with optional integration of chemoradiotherapy in selected cases [55]. FOLFIRINOX combines agents with complementary mechanisms of action: oxaliplatin induces platinum-DNA crosslinks, irinotecan inhibits topoisomerase I, 5-fluorouracil inhibits thymidylate synthase and interferes with RNA and DNA synthesis, and leucovorin enhances the cytotoxic effect of 5-fluorouracil by stabilizing its interaction with thymidylate synthase [55]. Although these approaches have improved outcomes in subsets of patients, treatment efficacy is frequently limited by the development of primary or acquired resistance, highlighting the need for predictive biomarkers that could support therapeutic decision-making.

Resistance in PDAC is not restricted to a single chemotherapeutic agent; however, gemcitabine resistance has been the most extensively investigated in the miRNA literature summarized in this section [56,57,58,59,60,61,62,63,64,65,66,67,68,69,70,71,72,73,74,75,76,77,78]. Resistance may also affect fluoropyrimidine-, platinum-, and irinotecan-containing regimens, including FOLFIRINOX. Tumor-related biological characteristics contributing to chemoresistance include hypoxia-associated signaling, cancer-associated fibroblast activity, extracellular vesicle-mediated communication, stemness-related pathways, epithelial–mesenchymal transition, altered DNA damage repair, and altered drug transport or metabolism [56,57,58,59,60,61,62,63,64,65,66,67,68,69,70,71,72,73,74,75,76,77,78].

A growing body of evidence implicates microRNAs in the modulation of gemcitabine sensitivity in PDAC. Overexpression of several miRNAs, including miR-155, miR-21, miR-31-5p, miR-140-3p, miR-146, miR-222-3p, miR-365, miR-342, miR-125a, miR-1246, miR-29a, miR-181b, miR-106b, miR-615-3p, miR-770-5p, miR-3178, and miR-6509-3p, has been associated with reduced gemcitabine sensitivity [56,57,58,59,60,61,62,63,64,65,66,67,68,69,70,71,72,73,74,75]. Conversely, downregulation of miRNAs such as miR-205, miR-7, let-7, miR-200, miR-217, and miR-101 has also been linked to chemoresistance [62,76,77,78]. Among these candidates, miR-21, miR-155, and miR-181b are the most consistently reported across independent studies, whereas many other miRNAs have been identified primarily in exploratory or preclinical analyses.

Importantly, the studies cited in this subsection were primarily designed to evaluate gemcitabine sensitivity, chemoresistance, extracellular vesicle-mediated signaling, or treatment-response mechanisms rather than stage-specific miRNA expression. As a result, the association of individual miRNAs with a specific pancreatic cancer stage cannot be consistently determined from the available evidence [56,57,58,59,60,61,62,63,64,65,66,67,68,69,70,72,73,74,75]. Accordingly, these miRNAs should be interpreted primarily as treatment-response or resistance-related candidates, not as validated stage-specific biomarkers.

Mechanistic insights into miRNA-mediated gemcitabine resistance have been provided by experimental studies. miR-155 was shown to suppress multiple tumor-suppressive target genes, including *TP53INP1*, thereby promoting chemoresistance [56]. In the same experimental context, gemcitabine exposure increased extracellular vesicle–mediated release of miR-155, suggesting a treatment-induced feedback mechanism that may further propagate resistance within the tumor microenvironment [56]. More broadly, miRNAs have been implicated in PDAC progression, metastasis, and tumor–microenvironment interactions, which may also contribute to therapy resistance [79]. These findings support gemcitabine-associated secretion of miR-155 in extracellular vesicles, rather than establishing a universal mechanism of miR-155 overexpression across all disease stages or clinical settings. Collectively, these findings support a biologically plausible role for miRNAs as modulators of chemotherapy response, although clinical validation remains limited.

Beyond chemotherapy, miRNAs have also been investigated in the context of radiotherapy response. The molecular basis of poor outcomes following chemoradiation was examined using both human tumor specimens and in vitro models. In resected tumors from patients who had received chemoradiotherapy, high c-Met expression was associated with significantly shorter overall and recurrence-free survival. Functional analyses revealed that radioresistant PDAC cell clones exhibited marked downregulation of miR-181b-5p, a miRNA that normally suppresses the transcription factor ETS1. Loss of miR-181b-5p resulted in ETS1-mediated upregulation of c-Met, thereby promoting an aggressive, radioresistant phenotype. These findings identify the miR-181b-5p–ETS1–c-Met axis as a potential prognostic marker of radiotherapy response and a candidate therapeutic target [80].

Additional miRNAs have been implicated in modulating radiotherapy sensitivity. Downregulation of miR-216b—a miRNA involved in FGFR1 signaling and EGFR/KRAS crosstalk—was associated with reduced radiosensitivity in PDAC patients lacking activating *KRAS* mutations [81]. In experimental models, miR-194-5p has been proposed to contribute to radioresistance by promoting tissue regeneration and angiogenesis while suppressing cell-cycle progression, potentially facilitating survival of tumor-repopulating cells after irradiation [82]. Other miRNAs linked to radioresistance include overexpressed miR-26a and miR-296-3p, as well as downregulated miR-181b-5p [80,83,84]. While these data underscore the complexity of miRNA-regulated radiation responses, most evidence remains preclinical.

With the emergence of targeted therapies for molecularly selected subsets of PDAC, miRNAs are also being explored as modulators of response to these agents. Although established molecular alterations such as germline or somatic defects in *BRCA1*, *BRCA2* and *PALB2*, mismatch-repair deficiency or microsatellite instability, *NTRK* or *RET* fusions, *BRAF V600E* alterations, and *KRAS G12C* mutations are not miRNA biomarkers, they provide the current clinical framework for molecularly guided therapy in PDAC and contextualize the still-experimental role of miRNA-based predictive markers. These alterations may inform the use of platinum-based chemotherapy, PARP inhibition, immune checkpoint inhibition, or selected targeted therapies in defined subgroups [85,86,87]. In particular, *KRAS G12C* represents a rare molecular subtype of PDAC, and *KRAS G12C* inhibitors are relevant only for this molecularly defined subgroup [85,86,87]. In routine practice, treatment resistance is generally inferred from disease progression during or after therapy, based on clinical assessment, imaging findings, and CA19-9 dynamics when informative. After progression, subsequent management depends on previous therapy, performance status, toxicity profile, and molecular findings, and may include alternative systemic regimens, molecularly targeted therapy in selected patients, clinical trial enrollment, or best supportive care [55,85,86,87]. Preclinical studies suggest that miRNAs may affect tumor cell sensitivity to selected targeted agents. For example, miR-130b-3p has been shown to enhance erlotinib responsiveness in PDAC cell lines by suppressing the EPHB4/JAK2/STAT3 signaling pathway [88]. Similarly, miR-124 inhibited *EPHA2* expression and increased erlotinib sensitivity in *KRAS*-mutant pancreatic cancer cells in vitro [89].

Overall, current evidence indicates that miRNAs may reflect treatment-relevant tumor biology in PDAC and could serve as predictive biomarkers of therapy response. However, most data supporting predictive utility derive from preclinical models or small clinical cohorts, and no miRNA-based assay is currently validated for guiding treatment selection in routine clinical practice. The selected microRNAs most consistently associated with treatment response, chemoresistance, or radiosensitivity in PDAC are summarized in Table 2.

## 4. Discussion

Circulating and fluid-derived miRNAs can discriminate PDAC from control conditions and, in selected settings, correlate with tumor stage and patient outcomes. Interpretation is limited by substantial heterogeneity in cohort composition, sample handling, analytical platforms, and normalization strategies, which reduces cross-study comparability and prevents identification of a single clinically transferable miRNA panel.

Overall, the reviewed evidence partially supports the working hypothesis that biological matrix selection strongly influences the diagnostic performance, reproducibility, and clinical applicability of microRNA-based liquid biopsy in PDAC. This hypothesis is supported by matrix-dependent differences in feasibility, biological specificity, and reproducibility, particularly between blood, urine, saliva, pancreatic cyst fluid, bile, and pancreatic juice. However, it cannot be fully confirmed because most available studies are small, heterogeneous, and lack prospective external validation.

CA19-9 remains the only routinely used biomarker in PDAC, but its diagnostic utility is limited, particularly in early-stage disease. Plasma- and serum-based miRNA panels frequently achieve diagnostic performance comparable to or exceeding CA19-9. However, evidence for early-stage PDAC detection remains limited and is based on small, heterogeneous cohorts without prospective validation. Differences in patient selection and control cohorts, particularly the inclusion of CP, contribute to variability across studies.

The choice of biological material is a key determinant of miRNA detectability and clinical applicability. Several miRNAs recur across more than one biological matrix, including miR-21, miR-1246, miR-196a/b, miR-210, miR-25, and members of the miR-200 family, as summarized in Table 1 and Table 3. However, none should be considered universally detectable or specific across all sample types because expression depends on the biological compartment, extraction method, analytical platform, and normalization strategy.

These findings suggest that no single biological matrix is universally optimal for miRNA-based liquid biopsy in PDAC. Instead, each sample type offers a different trade-off between accessibility, analytical reproducibility, and lesion proximity. Blood and urine appear most suitable for future non-invasive diagnostic validation, whereas pancreatic cyst fluid, bile, and pancreatic juice may be more relevant for adjunctive risk stratification in selected patients undergoing endoscopic evaluation. In some blood-based studies, circulating miRNA panels achieved diagnostic performance comparable to or higher than CA19-9, although these findings require external validation [16]. However, blood-based matrices have several limitations. miRNA profiles may differ between serum and plasma samples, partly because coagulation-related cell lysis during serum preparation can release additional RNAs and influence measured concentrations [15]. Blood-derived miRNAs reflect a heterogeneous systemic pool rather than direct tumor-derived signals, which reduces biological specificity compared with lesion-proximal fluids [51]. Additional challenges include pre-analytical variability, normalization differences, and small study cohorts [16]. Salivary miRNA profiles are inconsistent and currently lack reproducibility, limiting their clinical applicability. These matrix-specific differences support the need for separate validation pathways rather than direct extrapolation of miRNA performance across biological materials.

Selected diagnostic miRNAs with the strongest or most repeatedly reported evidence across biological matrices are summarized in Table 3.

Selected miRNAs with reported prognostic relevance are summarized in Table 4.

Among these candidates, miR-210/miR-210-3p deserves cautious interpretation, as it is a hypoxia-associated miRNA implicated in multiple human disorders and cancer types; therefore, its biological relevance appears more closely linked to hypoxic stress, angiogenesis, invasion, and treatment resistance than to pancreas-specific tumor origin [93].

As summarized in Table 2, Table 3 and Table 4, several miRNAs recur across independent studies and biological matrices, but their clinical meaning differs depending on context. Some candidates, such as miR-25, miR-1246, miR-196a/b, miR-200b, and miR-21, are mainly discussed in relation to diagnostic discrimination, whereas others, including miR-19a-3p, miR-210, and miR-181b-5p, appear more closely related to prognosis, treatment response, or therapy resistance. Mechanistically, these candidates may reflect biologically relevant processes, including epithelial differentiation, hypoxia-related signaling, stemness, extracellular vesicle-mediated communication, and tumor-suppressor pathway dysregulation. Importantly, none of these candidates is sufficiently specific, reproducible, or clinically validated to function as a standalone biomarker. Their performance depends on the biological material, control cohort, analytical method, normalization strategy, and clinical endpoint. Therefore, current evidence supports the development of standardized multi-marker panels rather than reliance on individual miRNAs.

Most studies focus on free-circulating miRNAs because this approach is technically simpler and more feasible than extracellular vesicle isolation. These studies consistently demonstrate associations between circulating miRNA expression and PDAC presence, stage, or survival. Exosome-associated miRNAs offer biological advantages, including increased stability, protection from enzymatic degradation, and selective cargo packaging, and may therefore provide more compartment-specific signals. However, their additional clinical value over free-circulating miRNAs remains unproven in prospective settings.

The main limitation of the current evidence is the poor reproducibility of miRNA panels across studies and biofluids. Panel composition varies substantially, and most studies are based on small cohorts without external validation, increasing the risk of false-positive findings. Additional sources of variability include differences in patient selection, comparison cohorts, sample handling, extraction methods, analytical platforms, and normalization strategies. These factors currently prevent the identification of a single clinically transferable miRNA panel.

From a clinical perspective, miRNA-based assays should be viewed as complementary tools rather than replacements for established diagnostic approaches. Integration with CA19-9 appears to be the most realistic near-term application, particularly in diagnostically challenging scenarios such as differentiation between PDAC and CP. Longitudinal monitoring remains promising but requires validation in well-defined clinical cohorts.

Future studies should prioritize prospective multicenter validation of a limited number of predefined miRNA panels, standardized workflows, and added value within defined clinical pathways. Particular attention should be given to clinically relevant high-risk populations, including individuals with hereditary pancreatitis, genetic cancer predisposition syndromes and new-onset diabetes. In addition, genetically determined susceptibility loci identified in genomic studies may help refine future risk stratification approaches [94]. A major unmet clinical need remains the development of biomarkers capable of detecting preinvasive pancreatic lesions, particularly PanIN, which are not detectable using current imaging modalities. Addressing this gap will be essential for improving early detection and long-term outcomes in PDAC.

## 5. Conclusions

Liquid biopsy-derived miRNAs represent promising complementary biomarkers for PDAC detection, risk stratification, and treatment-response assessment. The available evidence partially supports the working hypothesis that biomarker performance depends strongly on the biological matrix analyzed, with serum and plasma offering the greatest clinical feasibility, urine showing promising non-invasive diagnostic potential, and cyst fluid, bile, and pancreatic juice providing higher lesion proximity but limited applicability because of invasive sampling. At present, no single miRNA or miRNA panel is sufficiently validated for standalone clinical implementation. Further work should prioritize standardized workflows, prospective multicenter validation, integration with CA19-9, and evaluation in well-defined high-risk populations. The field should move beyond discovery-oriented candidate lists toward predefined, matrix-specific microRNA panels evaluated prospectively against clinically relevant diagnostic comparators. Such integrated approaches represent the most realistic path toward clinical translation.

## Figures and Tables

**Table 1:** Sources of biological material for miRNA-based liquid biopsy in PDAC.

Material	Key Advantages	Key Limitations	References
Serum	Easily accessible; compatible with routine clinical workflows; extensive clinical evidence; relatively stable circulating miRNAs	Hemolysis-related bias; limited tumor specificity due to systemic origin	[15,19,20,53]
Plasma	Lower hemolysis compared with serum; reproducible profiles; suitable for early-stage detection	Anticoagulant-related variability; limited organ specificity	[16,17,54]
Urine	Fully non-invasive; suitable for repeated sampling; low protein background facilitating analysis	Indirect biological link to pancreas; influenced by hydration and pH	[21,23,24,25]
Saliva	Completely non-invasive; convenient for screening applications	High biological variability; low pancreas specificity; limited reproducibility	[26,27,28,29]
Stool	Non-invasive; reflects pancreatic exocrine secretion and tumor–gut interaction	Low abundance of pancreatic miRNAs; high variability; microbiome interference	[47,48,49]
Pancreatic cyst fluid	High lesion specificity; direct contact with neoplastic epithelium; useful for IPMN/MCN stratification	Invasive acquisition (EUS-FNA); not suitable for screening	[35,37,39,40]
Bile/pancreatic juice	High lesion proximity; enriched tumor-derived signals	Invasive procedures (ERCP/EUS); enzymatic degradation; limited to selected indications	[50,51,52]

**Table 2:** Selected miRNAs associated with treatment response and therapy resistance in PDAC.

miRNA	Therapy Context	Key Findings	Biological Mechanism	Potential Clinical Application	References
miR-21	Gemcitabine	Overexpression associated with chemoresistance	Anti-apoptotic signaling and oncogenic pathways	Prediction of poor chemotherapy response	[57,58]
miR-155	Gemcitabine	Promotes resistance via *TP53INP1* suppression	Tumor–stroma interaction and exosome-mediated resistance	Therapy stratification and resistance monitoring	[56]
miR-181b-5p	Radiotherapy	Downregulated in radioresistant tumors	ETS1–c-Met signaling axis	Prediction of radiotherapy response	[80]
miR-34a	Chemotherapy, targeted therapy	Altered expression linked to TP53 pathway	Tumor suppressor regulating apoptosis and cell cycle	Therapeutic targeting and sensitivity modulation	[90]
miR-216b	Radiotherapy	Downregulation associated with reduced radiosensitivity	EGFR/KRAS-related signaling pathways	Treatment optimization	[81]
miR-1246	Chemotherapy	Overexpression associated with chemoresistance	Stemness-related pathways and survival signaling	Predictive biomarker for treatment response	[67]

**Table 3:** Selected diagnostic miRNA candidates reported in PDAC liquid biopsy studies.

miRNA	Biological Material	Key Findings	Biological/Clinical Significance	Potential Clinical Impact	References
miR-25	Serum	High diagnostic accuracy in large PDAC cohorts (AUC ~0.91)	Promotes proliferation and tumor growth	Non-invasive diagnostic adjunct with CA19-9	[19]
miR-1246	Urine, Serum	Consistently upregulated across biofluids	Associated with aggressiveness and stemness	Non-invasive detection with early-stage potential	[23,24]
miR-196a/b	Serum, Stool	Strong discrimination of PDAC and high-grade PanIN	Marker of high-risk precursor lesions	Early detection and surveillance	[47,91]
miR-200b	Pancreatic cyst fluid	Differentiates mucinous from non-mucinous cysts	Reflects epithelial differentiation	Improved cyst stratification	[35]
miR-3940-5p/miR-8069	Urine (exosomes)	Elevated in urine, low in serum	Fluid-specific exosomal transport	Diagnostic panels with CA19-9	[24]
miR-143/miR-155/miR-216a	Stool	Detectable with differential expression	Reflect pancreatic-gut interaction	Non-invasive adjunct screening	[48,49]
miR-3679-5p/miR-940	Saliva	Dysregulated with high sensitivity, low specificity	Low pancreas specificity	Exploratory screening	[29]

**Table 4:** Selected miRNA candidates with reported prognostic relevance in PDAC.

miRNA	Associated Outcome	Key Findings	Biological Significance	Clinical Implication	References
miR-19a-3p	Overall survival	Elevated levels associated with shorter OS	Oncogenic signaling	High-risk patient identification	[20]
miR-210	Survival, aggressiveness	Upregulated in hypoxia, worse outcomes	Hypoxia-driven phenotype	Prognostic biomarker	[92,93]
miR-1246	Stage, aggressiveness	Higher expression in advanced disease	Stemness and invasion	Prognostic enrichment	[23]
miR-22-3p/miR-642b-3p/miR-885-5p	Tumor stage	Expression correlates with disease stage	Tumor progression	Disease stratification	[18]

## Data Availability

No new data were created or analyzed in this study. Data sharing is not applicable to this article.
